# Supplementary material for: A Metabolomic Approach and Traditional Physical Assessments to Compare U22 Soccer Players According to Their Competitive Level
Source: Biology (Basel). 2022 Jul 25;11(8):1103. doi: 10.3390/biology11081103 (PMC9331507; doi:10.3390/biology11081103)
Supplement: Supplementary file 1 [file biology-11-01103-s001.zip › biology-1792531-supplementary.pdf]

Supplementary Materials for

A Metabolomic Approach and Classical Physical Assessments to Compare U22 Soccer Players According to Their Competitive Level

João P. da Cruz*, Fábio N. dos Santos, Felipe M. Rasteiro, Anita B. Marostegan, Fúlvia B. Manchado-Gobatto, Claudio A. Gobatto.

*Corresponding author. Email: jpdacruz97@hotmail.com

This PDF file includes:

Supplementary table 1

Supplementary table 2

Supplementary figure 1

Supplementary figure 2

Supplementary table 1. Differentiated features of the physiological profiles of soccer players from both elite and non-elite teams for the ESI (+) mode by fold-change analysis with threshold 2.

	ESI(+)	
**Features**	**Fold Change**	**log2(FC)**
M404T1_3	0.066231	-3.9164
M755T3	0.22876	-2.1281
M740T4	0.27924	-1.8404
M1272T8	3.5576	1.8309
M514T8	0.29087	-1.7816
M974T5_2	3.3745	1.7547
M660T2	3.1414	1.6514
M443T2_2	0.32328	-1.6292
M747T4_1	0.33368	-1.5834
M243T2	2.8692	1.5206
M721T2	0.35467	-1.4955
M605T5	2.8189	1.4951
M901T7_1	2.6509	1.4065
M316T0_1	2.6448	1.4032
M463T1_1	2.6396	1.4003
M715T4_2	2.5922	1.3742
M676T2_2	2.5366	1.3429
M774T5_2	2.5064	1.3256
M554T7_1	2.4824	1.3118
M291T1_1	2.4544	1.2954
M532T1_1	2.4439	1.2892
M638T2	2.4179	1.2738
M932T2_2	0.4143	-1.2713
M829T5	2.407	1.2672
M909T2	0.41725	-1.261
M340T0_2	2.3884	1.256
M370T0	2.3641	1.2413
M726T1	0.431	-1.2142
M784T5_3	2.3152	1.2112
M359T1_1	2.2792	1.1885
M761T5_1	2.2739	1.1852
M286T3	0.44056	-1.1826
M209T2_2	0.44244	-1.1764
M763T5_1	0.44428	-1.1704
M820T4	2.25	1.1699
M583T10	2.2305	1.1574
M406T1_2	2.2258	1.1543
M204T2	2.2248	1.1537
M787T9	0.45391	-1.1395
M238T0_4	2.1746	1.1208
M765T4_2	2.1721	1.1191
M238T0_3	2.161	1.1117
M313T1_2	2.1585	1.11
M145T0_2	2.1557	1.1082
M369T1_2	2.1525	1.106
M1087T1_1	0.46624	-1.1008
M172T20_2	2.1078	1.0757
M334T1_1	2.1074	1.0754
M375T1_2	0.47463	-1.0751
M628T7	0.47489	-1.0743
M161T15_2	0.47661	-1.0691
M889T10_3	2.0904	1.0638
M591T8	0.4794	-1.0607
M773T5_3	2.0767	1.0543
M635T2_2	2.0741	1.0525
M293T1	0.48406	-1.0467
M1214T7_1	2.0541	1.0385
M909T10_1	2.0535	1.0381
M918T10_3	2.0514	1.0366
M701T7	2.0406	1.029
M414T1_4	2.0387	1.0277
M998T10	0.49106	-1.026
M650T8_1	2.0329	1.0236
M677T2_2	2.0316	1.0226
M356T0	2.0297	1.0213
M887T9_1	2.0292	1.0209
M591T1	0.49313	-1.0199
M812T5_3	0.4932	-1.0198
M527T1_2	0.49432	-1.0165
M906T10_1	0.49455	-1.0158
M917T9_1	2.0216	1.0155
M417T2_1	0.49624	-1.0109
M705T12	2.0117	1.0084
M103T19_2	2.008	1.0058
M698T5	2.0005	1.0004
M856T7_1	0.49993	-1.0002

Supplementary table 2. Differentiated features of the physiological profiles of soccer players from both elite and non-elite teams for the ESI (-) mode by fold-change analysis with threshold 2.

	ESI(-)	
**Features**	**Fold Change**	**log2(FC)**
M798T6	0.13076	-2.935
M348T1_2	4.5819	2.196
M583T23_1	4.1967	2.0693
M924T23	3.546	1.8262
M187T27	3.4989	1.8069
M608T1_3	0.29925	-1.7406
M495T1	0.31587	-1.6626
M682T3	3.1162	1.6398
M571T21	0.38401	-1.3808
M465T3_2	0.38648	-1.3715
M671T3	2.5728	1.3633
M263T1_1	2.5546	1.3531
M887T7_3	2.5442	1.3472
M777T7_2	2.524	1.3357
M993T9	0.39713	-1.3323
M961T23	2.4898	1.316
M789T6_2	2.4542	1.2952
M773T6	0.40775	-1.2942
M580T1_3	0.40804	-1.2932
M698T3	2.4483	1.2918
M300T1_2	2.3832	1.2529
M966T7	0.42145	-1.2466
M1005T23_2	2.3676	1.2434
M449T0	0.42553	-1.2327
M949T7_1	0.43393	-1.2045
M620T2	2.2954	1.1987
M267T1_1	2.286	1.1928
M447T1_1	0.44629	-1.1639
M803T7_1	0.44682	-1.1622
M349T1_2	2.2118	1.1452
M954T8	2.2051	1.1408
M857T6	2.1953	1.1344
M889T7	0.45985	-1.1207
M846T23	2.1735	1.12
M338T1_4	0.46173	-1.1149
M397T3_2	2.1594	1.1107
M734T23	2.1467	1.1021
M455T1_2	0.46588	-1.102
M483T23_1	2.1342	1.0937
M797T7_2	2.1257	1.0879
M724T7	2.1188	1.0833
M682T8	0.47306	-1.0799
M439T1_1	2.1135	1.0796
M373T25_1	0.47326	-1.0793
M1137T6	2.1006	1.0708
M539T2	2.078	1.0552
M530T23	2.0583	1.0414
M499T1	2.057	1.0406
M335T0	0.48729	-1.0372
M752T7_2	0.4874	-1.0368
M827T6_2	2.0507	1.0361
M369T1_4	0.48771	-1.0359
M247T0_1	2.0499	1.0355
M833T23	0.48808	-1.0348
M791T6_2	2.0452	1.0322
M409T3	2.0434	1.031
M424T2	2.0419	1.0299
M558T1_1	0.48986	-1.0295
M545T23_2	2.0269	1.0193
M383T0_1	2.0266	1.0191
M1217T23	2.0172	1.0123
M223T0_2	0.49677	-1.0094
M884T8	2.0053	1.0038
M829T6_1	0.49881	-1.0034

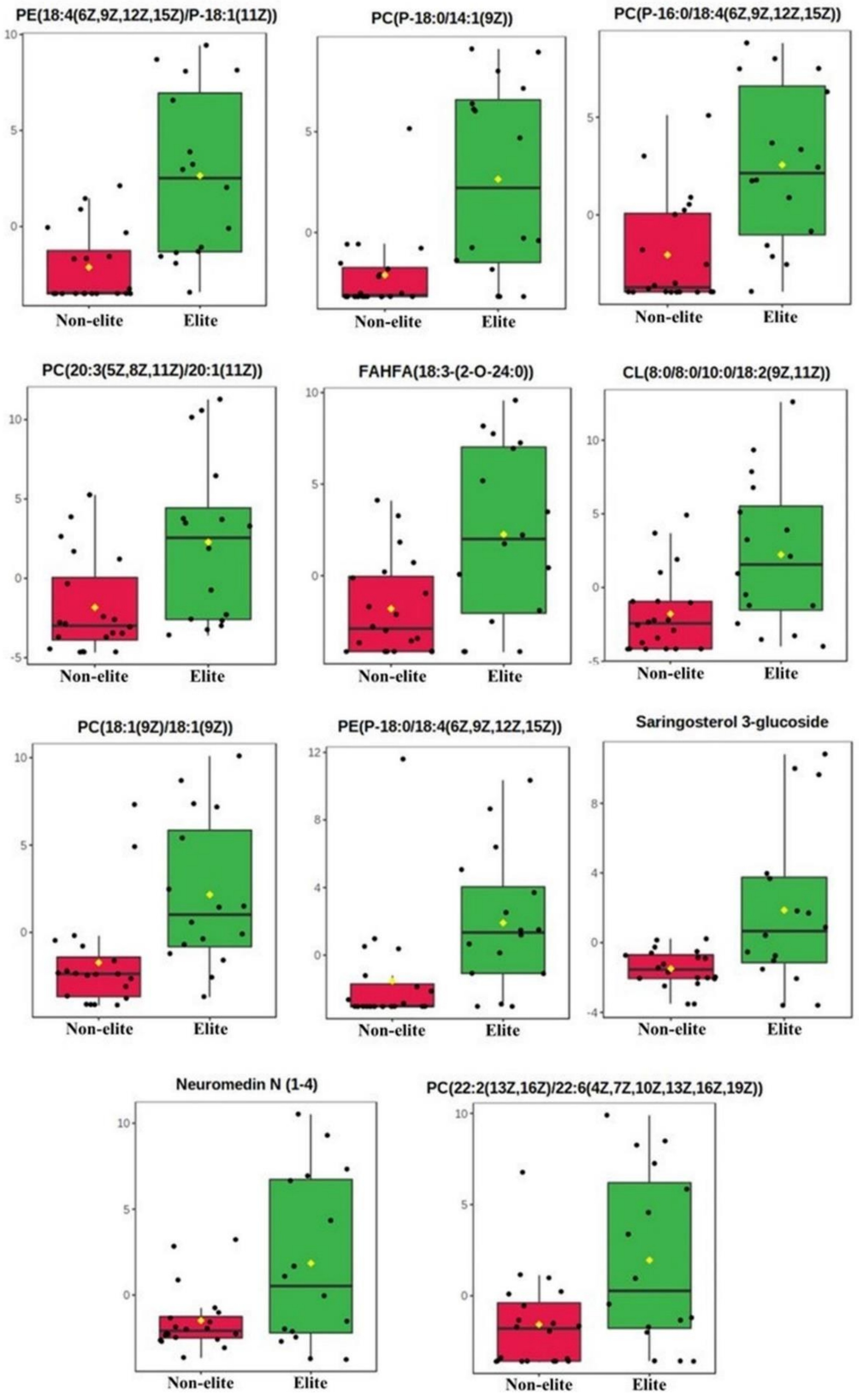

Supplementary figure 1. Relative abundances in the mean and standard deviation of the corresponding putative metabolites in each team for the ESI (+) mode. All metabolites are statistically different by fold-change analysis with threshold 2.

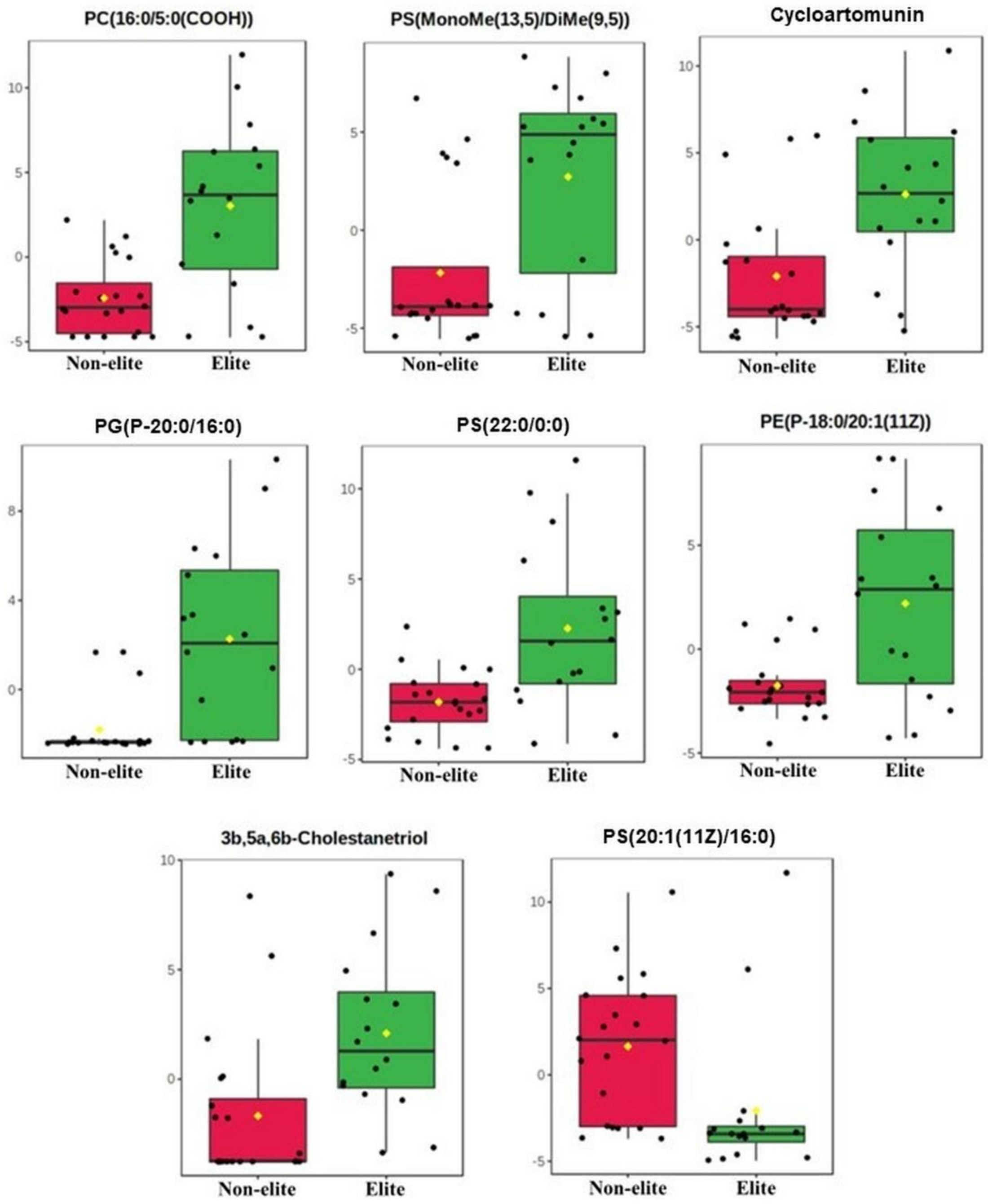

Supplementary figure 2. Relative abundances in the mean and standard deviation of the corresponding putative metabolites in each team for the ESI (-) mode. All metabolites are statistically different by fold-change analysis with threshold 2.
